# Joint Optimization of Antenna System Matching and Specific Absorption Rate Focusing in Microwave Hyperthermia Cancer Treatment

**DOI:** 10.3390/cancers17030386

**Published:** 2025-01-24

**Authors:** Maryam Firuzalizadeh, Rossella Gaffoglio, Giorgio Giordanengo, Marco Righero, Marcello Zucchi, Giuseppe Musacchio Adorisio, Aurora Bellone, Alberto Vallan, Guido Perrone, Giuseppe Vecchi

**Affiliations:** 1 Department of Electronics and Telecommunications, Politecnico di Torino, 10129 Turin, Italy; maryam.firuzalizadeh@polito.it (M.F.); marcello.zucchi@polito.it (M.Z.); aurora.bellone@polito.it (A.B.); alberto.vallan@polito.it (A.V.); guido.perrone@polito.it (G.P.); 2 Advanced Computing, Photonics & Electromagnetics (CPE) Area, Fondazione LINKS, 10138 Turin, Italy; rossella.gaffoglio@linksfoundation.com (R.G.); giorgio.giordanengo@linksfoundation.com (G.G.); marco.righero@linksfoundation.com (M.R.); giuseppe.musacchio@linksfoundation.com (G.M.A.)

**Keywords:** active reflection coefficient, antenna arrays, cost function, hyperthermia treatment (HT), optimization, specific absorption rate (SAR)

## Abstract

Objective: Microwave hyperthermia is a clinically proven cancer treatment used in combination with conventional therapies to enhance the overall treatment outcome. It consists in selectively increasing the temperature of tumor cells to 40–44 °C by means of electromagnetic fields that are externally generated and coupled to the patient body via antenna applicators. The primary goal is to shape the power deposition (specific absorption rate, SAR) with focusing on the tumor region, and minimizing the risk of hotspots in the surrounding healthy tissues. Methods: For non-superficial tumors, phased-array antennas are used to focus the energy on the tumor. Finding patient-specific optimal antenna feeding coefficients represents an essential step to ensure an effective and safe administration of the heating. In this article, we present a way to optimize the array power transfer effectiveness (impedance matching) that does not deteriorate the spatial power deposition performance. A global optimization approach is adopted, using a cost function properly tailored to incorporate the active reflection coefficients of the array and the Hotspot-to-Target SAR Quotient (HTQ)—the latter being the standard in hyperthermia applications. Results: The effectiveness of the technique is demonstrated in a scenario relevant to the treatment of tumors in the neck region. The results show that our method significantly improves antenna matching without compromising the HTQ, achieving values within the recommended limits. The performance of the proposed approach is also experimentally tested with full heating in a corresponding phantom. Conclusions: This study introduces an optimization approach that enhances phased-array antenna performance for hyperthermia treatments without affecting spatial power deposition.

## Simple Summary

Microwave hyperthermia is a cancer treatment that enhances the effectiveness of chemotherapy and radiotherapy by selectively heating tumor cells to the temperature range of 40–44 °C by means of antenna systems. In this paper, we focus on improving the effectiveness of phased-array antenna applicators, which are used to target deep-seated and sub-superficial tumors. Our method optimizes the power transfer from the antennas to the tumor while maintaining precise energy deposition in the tumor region and minimizing risks to surrounding healthy tissues. The proposed approach significantly improves the performance of the antenna system while ensuring an efficient and safe administration of the heating.

## 1. Introduction

Microwave hyperthermia (HT) is a clinically proven, promising approach to cancer treatment. It consists of selectively heating tumor cells to temperatures between 40 and 44 °C [1]. The underlying mechanism of hyperthermia involves transferring energy to the tissue through the absorption of microwave radiation, which causes molecular motion and consequently heat generation [2,3]. The localized heating improves the penetration of chemotherapy drugs into the tumor cells, and increases the sensitivity to radiation, making HT a potent sensitizer for chemotherapy and radiotherapy. Several clinical trials have demonstrated how the use of HT in combination with conventional cancer therapies significantly enhances tumor response and improves patient survival rates [4,5,6,7,8,9,10].

To effectively treat sub-superficial and deep-seated tumors, the primarily applicators used involve phased-array antenna systems [11,12] equipped with a waterbolus—a bag containing circulating water maintained at a constant temperature of 20–25 °C—used clinically to prevent skin overheating and facilitate electromagnetic coupling to the body [13].

In clinical practice, a preliminary hyperthermia treatment planning (HTP) step is prescribed by current guidelines [14] to determine the set of optimal steering parameters (amplitude and phase) for each antenna in the applicator array [15]. Patient-specific numerical simulations are employed in state-of-the-art clinical approaches [16], involving electromagnetic simulations of the segmented model of the patient’s region of interest (ROI) obtained from CT or MRI scans [17], as well as the geometry of the applicator. Central to all optimization techniques described in the literature is the optimization of the antenna feedings to maximize power deposition within the tumor region while minimizing the occurrence of hotspots in the surrounding healthy tissues.

### 1.1. State of the Art

The actual medium in which the array operates is patient specific, and hence different in all applications. Hence, the antennas are usually designed to operate in an average situation, which typically corresponds to the coupling medium alone in the entire region of interest (e.g., a waterbolus [18] for deep-seated tumors). The antenna geometry and array layout are designed to have low reflection ($S_{ii}$) at each port and low inter-element coupling ($S_{ij}$) in that reference situation.

Current optimization techniques employed in clinical settings are either temperature based [19] or SAR based [20,21,22]. The temperature-based approach aims to directly optimize temperature distribution within the tumor and in the surrounding tissues, yet the outcome can be affected by the uncertainty characterizing thermal parameters [23,24]. Conversely, SAR-based optimization techniques exploit the SAR as a surrogate metric as it correlates strongly with the temperature increase and exhibits lower computational complexity while yielding favorable treatment outcomes [25]. Both SAR- and temperature-based methods are currently in use in the clinical setting and both require real-time adjustments during treatment [20,26]. SAR-based optimization is favored for its efficiency, and will be the focus of this study.

### 1.2. Innovation

State-of-the-art array coefficient synthesis approaches optimize SAR (or temperature) distribution but do not consider the array impedance mismatching due to operating in a situation (patient) that differs from the array design environment.

The objective of this paper is to optimize the array antenna matching in the presence of a given patient medium, without affecting the SAR deposition profile performance. To the best of the authors’ knowledge, this has not been addressed yet in the existing literature.

The presented approach significantly improves antenna matching without compromising the Hotspot-to-Target SAR Quotient (HTQ). The proposed technique is demonstrated in an environment representative of HT in the neck region. The approach is first validated on an “in silico” (simulative) model of the setup, then by an experimental validation using a physical phantom.

The proposed method has general applicability to sub-superficial and deep-seated tumors located in all regions of the human body that (intrinsically) require the use of phased-array applicators for energy focusing. In our present application, we will focus on deep-seated tumors in the H&N region; the underlying microwave HT system has been demonstrated in other studies for several other anatomical districts [1,18,27].

## 2. Joint Optimization Approach

The primary objective of SAR-based optimization is to maximize power deposition within the tumor region while minimizing the risk of overheating in the surrounding healthy tissues. Here, we recall the definition of the specific absorption rate (SAR):

(1) $$SAR\left(r\right)=\frac{\sigma (r)}{2\rho (r)}{\left|E\left(r\right)\right|}^{2}$$

where σ (S/m) is the electrical conductivity, ρ (kg/m^3^) is the tissue mass density, and E (V/m) represents the total electric field (using the peak value convention), all evaluated at position vector r. The total electric field can be expressed as a superposition of the electric fields generated by each antenna of the *N*-element array acting independently and the unknown excitation coefficients, i.e.,

(2) $$E\left(r\right)=\sum_{n=1}^{N}{\tilde{\nu }}_{n}\;e_{n}\left(r\right),$$

where $e_{n}\left(r\right)$ is the field generated by the *n*th antenna when fed by unitary excitation, and ${\tilde{\nu }}_{n}$ is the *n*th antenna excitation coefficient—considering both amplitude and phase—as part of the array, which can be conveniently expressed as follows:

(3) $${\tilde{\nu }}_{n}=\nu_{0}\xi_{n}e^{i\varphi_{n}},$$

where $\varphi_{n}\in \left[0,2\pi \right)$ represents the phase, $\xi_{n}\in \left[0,1\right]$ denotes the normalized amplitude coefficient, and $\nu_{0}=\sqrt{2R_{0}P_{0}}/{∥\xi ∥}_{2}$ is a constant amplitude coefficient, with $R_{0}=50$ Ω being the reference impedance of each antenna, $P_{0}$ the total power delivered to the array, and ${∥\;\cdot \;∥}_{2}$ the Euclidean norm. Note that the fields $e_{n}\left(r\right)$ in (2) are obtained in the actual situation, i.e., considering the actual scenario (patient and applicator), and are the extension of the embedded pattern concept [28].

The standard cost function to be minimized in SAR-based optimization is the Hotspot-to-Target SAR Quotient (HTQ) [20], defined as follows:

(4) $$\mathrm{HTQ}=\frac{\langle {\mathrm{SAR}}_{\;V1}\rangle }{\langle {\mathrm{SAR}}_{\;\mathrm{TARGET}}\rangle },$$

where $\langle {\mathrm{SAR}}_{\;V1}\rangle$ is the average SAR in V1, with V1 being 1% of the healthy volume with the highest SAR [13,29], and $\langle {\mathrm{SAR}}_{\;\mathrm{TARGET}}\rangle$ the average SAR in the target region. While the goal of standard SAR-based optimization is to minimize the HTQ, reaching a value of HTQ≤1 is considered acceptable in clinical settings [30].

Antenna array matching is described by the *active reflection coefficients* $\Gamma_{n}^{a}$ at the antenna ports [28,31]:

(5) $$\Gamma_{n}^{a}=S_{nn}+\sum_{m\neq n}S_{nm}\frac{{\tilde{\nu }}_{m}}{{\tilde{\nu }}_{n}},$$

where *S* is the scattering matrix, and ${\tilde{\nu }}_{1},\ldots ,{\tilde{\nu }}_{N}$ are the considered excitation coefficients. Throughout this paper, the magnitude of the reflection coefficient will be expressed in dB, as is common in the related literature, i.e., 20log|Γ| is intended when reporting the value in dB.

In this article, we propose an optimization method that includes active reflection coefficients bounding into the cost function. It is apparent that array matching imposes additional constraints to the optimization; hence, SAR performance is expected to be affected with respect to the case in which no such constraints are enforced. Hence, it is recognized that the absolute minimization of active reflection coefficients is not the best strategy. It is instead more expedient to require that the active reflection coefficients are lower than a given threshold (a typically acceptable threshold is $\Gamma_{th}=-10$ dB). To achieve this joint optimization, we define the cost function F as follows:

(6) $$F=\alpha \;\mathrm{HTQ}+\left(1-\alpha \right)\sum_{n=1}^{N}f_{n},\;f_{n}=\left\{\begin{array}{cc} 0 & \mathrm{if}\;\left|\Gamma_{n}^{a}\right|\leq \Gamma_{th}\\ 1/N & \mathrm{if}\;\left|\Gamma_{n}^{a}\right|>\Gamma_{th} \end{array}\right.$$

Here, α is a weighting factor and $\Gamma_{th}$ is a threshold value.

For the optimization, we employ a particle swarm optimization (PSO) algorithm [32,33]. We find that α=0.5 is the best choice, consistent with the fact that, after a few iterations, the HTQ is in the order of 1.

## 3. Examples of Application

### 3.1. Reference Testbed

**Figure 1:**
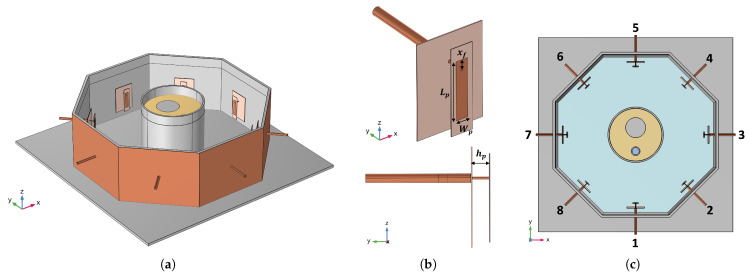
(**a**) In silico model of the mock-up of the HT applicator. (**b**) Employed antenna and optimized dimensions. (**c**) Top view of the HT mock-up. The reported numbers are used to index the antennas of the array.

The testbed considered for the verification of the proposed procedure is a mock-up reproducing a typical HT applicator used for treating deep-seated and sub-superficial tumors in the head and neck (H&N) region [18,27]. The in silico model of this mock-up, illustrated in Figure 1, was implemented in the simulation software COMSOL Multiphysics [34]. The dielectric and thermal properties of the materials used in this mock-up are detailed in Table 1.

The HT applicator (see Figure 1a) consists of a circular array of eight patch antennas immersed in water, and is arranged in an octagonal Poly(methyl methacrylate) (PMMA) container with a circumradius of 20 cm and height of 12 cm. A PMMA hollow cylinder (outer diameter = 12.4 cm, inner diameter = 11.8 cm, height = 12 cm) simulates the neck of a patient using a phantom that mimics the dielectric properties of human muscle [35]. It contains one solid and one hollow PMMA cylinder, which simulate the spine and the trachea, respectively. The space between the container walls and the central phantom is filled with demineralized water, which serves as the substrate for the patch antennas and forms the so-called waterbolus [18].

The phantom used in this study is a semi-solid agar-based phantom, properly developed to reproduce the dielectric properties of muscle tissue (see Table 1) [36,37].

The patch antennas forming the array were designed to be matched at the operating frequency f=434 MHz [38]. This frequency lies within a commonly used ISM band, and it has been demonstrated to provide a good balance between tissue penetration and energy absorption and size of the applicators, thus making it well suited for deep-seated tumor HT [39,40]. To speed up the optimization of the antenna dimensions, a layered scenario was considered in CST Microwave Studio [41], where a single patch antenna is simulated in a water environment in front of finite dielectric layers of PMMA and muscle-mimicking material reproducing the neck phantom [38]. The distance between the patch and the neck phantom is fixed to 8 cm, nearly twice the value at which the reflection coefficient starts to exhibit a stable behavior. With reference to Figure 1b, the optimized antenna dimensions were found to be $L_{p}=33.85$ mm, $W_{p}=7.13$ mm, $h_{p}=9$ mm, and $x_{f}=5.66$ mm, the latter being the distance of the coaxial feed to the patch edge. Both the ground and the patch were printed on layers of I-Tera MT40 ($\varepsilon_{r}=3.45$, tanδ=0.0031). The distance between the antenna and the ground, $h_{p}$, was further numerically adjusted and experimentally fine-tuned to center the resonance at 434 MHz when the antenna is part of the array in the applicator. This resulted in increasing the value of $h_{p}$ to 10.5 mm.

**Figure 2:**
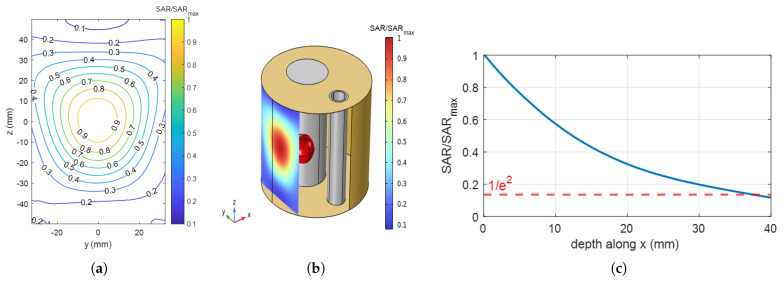
(**a**) Simulated normalized SAR contours at a depth of 1 cm within the phantom along the *x*-axis. The 0.5 isoline encloses the region used to estimate the effective field size (EFS). (**b**) Normalized SAR map visualized on a plane at 1 cm depth within the phantom. (**c**) Normalized SAR values versus the depth inside the phantom, evaluated along the *x*-axis for constant y=z=0 mm. All results refer to the case when only antenna 7 is fed (see Figure 1c for details on the numbering used).

To quantify the ability of the designed setup to couple the electromagnetic energy to the phantom, the effective field size (EFS), defined as the area enclosed by the 50% SAR curve at 1 cm depth within the tissue [42], and the penetration depth, defined as the depth at which the SAR becomes $1/e^{2}$ of its surface value [43], were simulated for antenna 7 (see Figure 1c) and are reported in Figure 2. Specifically, Figure 2a reports the simulated normalized SAR isolines at 1 cm depth within the tissue when only antenna 7 is fed (see the SAR map in Figure 2b), while Figure 2c shows the normalized SAR evaluated in the same configuration as a function of the depth inside the phantom along the central *x*-axis (0mm depth means the surface of the phantom). The estimated values for the EFS and the penetration depth were 21.49 cm^2^ and 37.32 mm, respectively.

**Figure 3:**
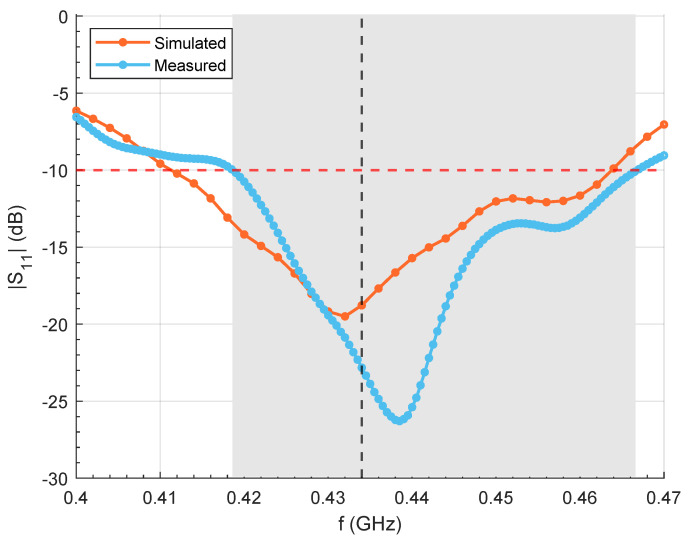
Reflection coefficient of a patch antenna used in the array simulated in COMSOL Multiphysics and measured in the experimental setup.

The simulated reflection coefficient of a single antenna (antenna 8 in Figure 1c) as part of the array applicator is reported in Figure 3 in comparison with the measured reflection coefficient obtained with the experimental realization of the HT setup. A good agreement was observed, achieving a bandwidth of 46 MHz around the central frequency (434 MHz) for the −10 dB threshold.

The considered target region is a sphere with radius $r_{t}=15$ mm located centrally within the neck phantom at spatial coordinates $\left(x_{t},y_{t},z_{t}\right)=\left(-30,-15,0\right)$ mm, with (0,0,0) being the coordinates of the center of the neck cylinder. In the performed optimizations, we decided to consider all phases as relative to antenna 1 (see Figure 1c), which means $\varphi_{1}=0^{{}^{\circ}}$, relative amplitude coefficients ($\xi_{n}$) are constrained to vary within 0.5 and 1, and the active reflection coefficient threshold $\left(\Gamma_{th}\right)$ in (6) is set to −10 dB.

The resulting optimized antenna coefficients extracted for both cost functions used in this study, i.e., HTQ and F, are reported in Table 2. Additionally, Table 2 presents the magnitude of the simulated active reflection coefficients $\left(\left|\Gamma_{n,sim}^{a}\right|\right)$ corresponding to each set of feeding coefficients.

The magnitude of the simulated active reflection coefficients listed in Table 2 highlight the mismatch issues associated with using the standard cost function (HTQ). Notably, except for the sixth antenna, all array elements exhibit active reflection coefficients higher than the specified threshold of $\Gamma_{th}=-10$ dB. In contrast, the magnitude of the simulated active reflection coefficients obtained using the proposed cost function $\left(F\right)$, as listed in the last column of Table 2, all fall below the −10 dB threshold since this criterion is directly incorporated into the proposed cost function $\left(F\right)$.

The optimization properties presented in Table 3 indicate that the final HTQ values for both optimization methods meet the typical threshold considered acceptable for clinical treatment—they are both less than 1. Moreover, there is no significant difference in computational demand between the two optimization methods.

**Figure 4:**
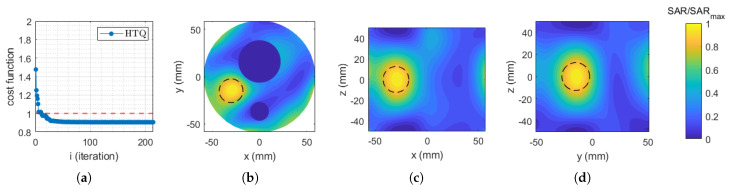
(**a**) Standard cost function (HTQ) evolution. Normalized SAR distribution obtained in COMSOL Multiphysics using the feeding coefficients corresponding to the minimization of the standard cost function (HTQ), displayed on the (**b**) xy plane, (**c**) xz plane, and (**d**) yz plane. The dashed circle indicates the profile of the considered target spherical region.

Figure 4a illustrates the convergence behavior of the PSO algorithm when minimizing the standard cost function (HTQ) across multiple iterations. The subsequent plots in Figure 4 depict the final normalized SAR distribution obtained in COMSOL Multiphysics using the optimized antenna coefficients presented in Table 2. These plots are displayed on three canonical planes passing through the target sphere at its centroid.

**Figure 5:**
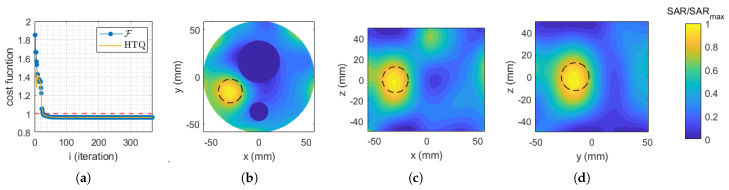
(**a**) Proposed cost function $\left(F\right)$ evolution alongside the corresponding HTQ. Normalized SAR distribution obtained in COMSOL Multiphysics using the feeding coefficients corresponding to the minimization of the proposed cost function (F), displayed on the (**b**) xy plane, (**c**) xz plane, and (**d**) yz plane. The dashed circle indicates the profile of the considered target spherical region.

Similarly, Figure 5a depicts the optimization process for minimizing the proposed cost function $\left(F\right)$ and standard HTQ over the PSO algorithm iterations. Using the optimized antenna coefficients obtained with the proposed approach (see Table 2), the subsequent plots shown in Figure 5 illustrate the final normalized SAR distribution generated in COMSOL Multiphysics, displayed on the three canonical planes intersecting the target sphere at its centroid.

### 3.2. Experimental Validation

**Figure 6:**
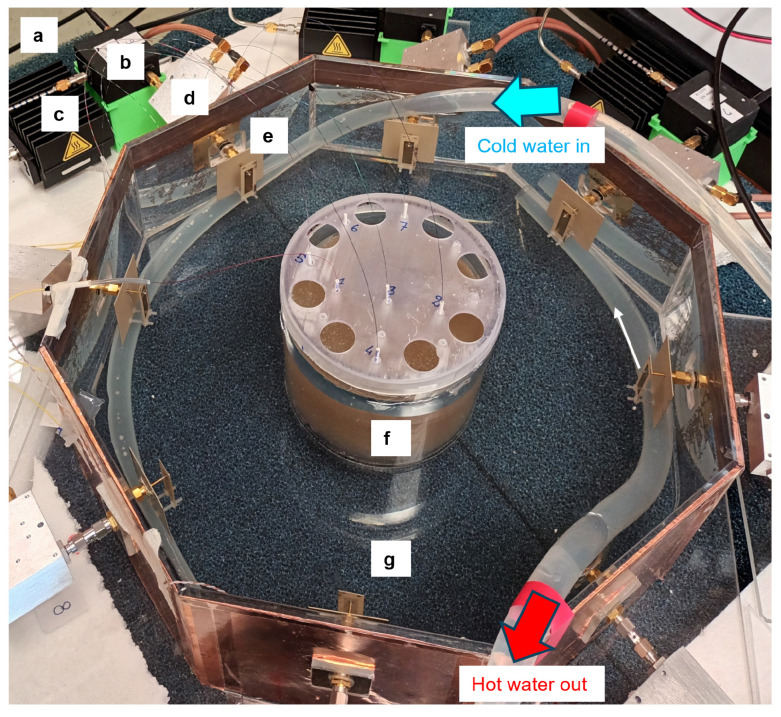
Setup of the experimental mock-up reproducing the HT applicator. Components are labeled as follows: (a) RF signal coming from Power Amplifier (PA)–Variable Gain Amplifier (VGA)–Phase Shifter (PS) chain, (b) circulator, (c) dummy load, (d) directional coupler, (e) patch antenna, (f) neck phantom, (g) waterbolus with circulating water.

The physical realization of the mock-up introduced in Section 3.1 is shown in Figure 6 in the active state along with the electronic setup with labeled components. This mock-up serves as a representative model of a typical HT applicator used for treating deep-seated and sub-superficial tumors in the H&N region [18,27]. As shown in Figure 6, the neck phantom cylinder filled with the agar-based phantom is placed at the center of the octagonal container reproducing the waterbolus and hosting the antenna array. The selected octagonal shape is a good trade-off between approximating a circular structure, having the needed number of antennas to focus the EM energy [18], and coping with manufacturing constraints.

**Figure 7:**
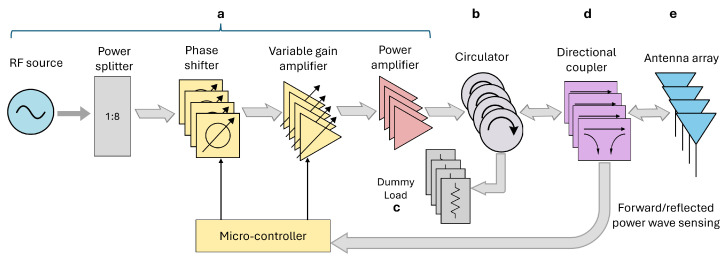
Block diagram of the antenna array feeding network. Arrows direction indicates the flow of RF power in the system. The alphabetical labels refer to Figure 6. Only 4 of the 8 channels are depicted to avoid confusion.

Figure 7 presents the block diagram of the feeding network, designed to deliver signals with stable amplitude and appropriate phase to the eight antennas of the applicator. The system starts with an RF signal generator (HP 8648A, Hewlett-Packard Company, Palo Alto, CA, USA) operating at 434 MHz and the signal is distributed to each antenna via a one-to-eight power splitter. For each channel, phase and amplitude adjustments are managed by a Phase Shifter (PS) and a Variable Gain Amplifier (VGA), both of which are controlled by a micro-controller. Then, the signal is amplified to a suitable power level by a Power Amplifier (PA) with a gain of 45 dB. The chain up to this point is labeled as (a) in Figure 6. A circulator with a dummy load is then incorporated to protect the PA from any reflected power wave from the antennas. Directional couplers are used to monitor both the forward and reflected power wave at the array ports, allowing the computation of active reflection coefficients for the feedback control.

**Figure 8:**
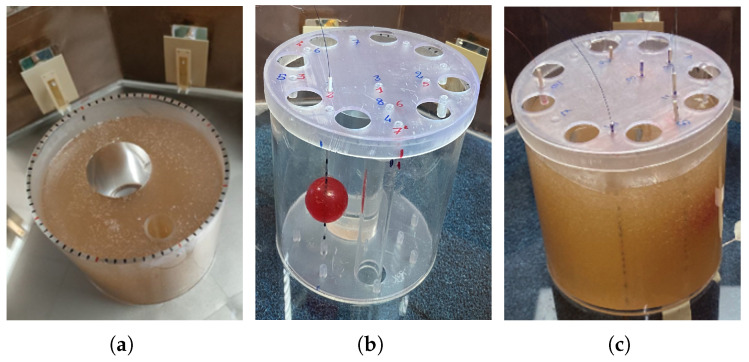
(**a**) View of the complete neck phantom in the experimental setup; (**b**) target sphere representing the tumor in its position inside the container with the system of FOS arrays; (**c**) agar-based phantom added to the neck container with the tumor inside.

The phantom used in this study replicates the dielectric properties of muscle tissue, as detailed in Table 1. This is achieved by modifying the recipe introduced in [36], creating a homogenized mixture of 90 °C demineralized water, salt, sugar, and agar–agar powder, and then pouring it into the neck-shaped container to solidify, as shown in Figure 8a. Figure 8b,c show the presence of the tumor target sphere, before and after the homogenized agar mixture is poured into the neck phantom, respectively. The relative permittivity ($\varepsilon_{r}$) and the electrical conductivity (σ) of the phantom listed in Table 1 are the result of weighted averages of independent measurements carried out using an open-ended coaxial probe and a commercial dielectric probe. For the thermal parameters, i.e., the thermal conductivity (*k*) and the heat capacity ($C_{p}$), the weighted averages reported in Table 1 were obtained from independent measurement sessions performed at 37 °C using standard methods and the Lasercomp FOX600 heat flux meter apparatus (TA Instruments, New Castle, DE, USA) [37,44].

**Figure 9:**
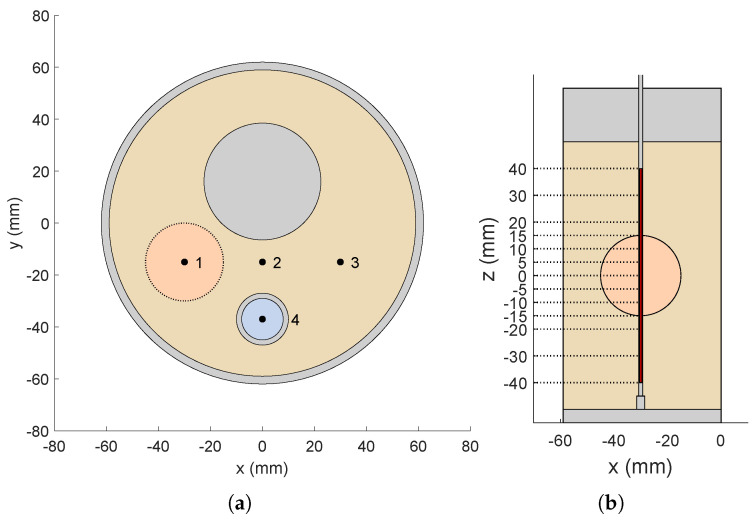
(**a**) Position of the FOS arrays on the xy plane. (**b**) Position of the FBG sensors on each array along the vertical (*z*-axis) direction.

To monitor temperature variations across the implemented mock-up in real time and without interfering with the electromagnetic field, dielectric temperature sensors were strategically positioned in four different locations, as illustrated in Figure 9a. Specifically, array 1 was positioned at the center of the tumor target sphere, while array 4 was inserted in the hollow cavity of the cylinder simulating the trachea. The sensors were Fiber Optic Sensors (FOSs) based on an array of Fiber Bragg Gratings (FBGs). The use of optical fibers makes the sensors intrinsically free from artifacts induced by the interaction with microwaves, such as self-heating effects. Moreover, their small size does not appreciably alter the temperature distribution. Among all the various types of fiber optic temperature sensors, FBG-based devices stand out for their unique combination of properties, such as a well-consolidated and reproducible fabrication, fast response, and robustness to noise [45]. Moreover, they are the only type that can be easily multiplexed along the same fiber to form several sensing points, obtaining a dense array of temperature sensors with good accuracy and reasonable cost. FBGs have already been proven to be well suited for real-time temperature monitoring during medical thermal treatments [46]. However, FBGs—like other FOSs—are also sensitive to mechanical deformations. Therefore, to mitigate this cross-sensitivity, the fiber containing the FBGs was enclosed in a small glass capillary sealed with epoxy. The final embodiment of the multipoint sensor was a small glass cylinder about 10 cm long, with an external diameter of 2 mm, embedding 13 sensing points with variable spacing from 5 mm to 10 mm (see Figure 9b) to provide the best spatial resolution where the maximum thermal gradient is expected. The small dimensions of each multipoint sensor (volume of about 0.3 cm^3^) have negligible perturbation effects both on the electromagnetic field and on the temperature distributions [46]. Each multipoint sensor was calibrated against a traceable thermometer in the temperature range from 15 °C to 45 °C. After the calibration, the deviation from the fitting model was found to be below 0.1 °C [47].

The SAR-based optimization process for the implemented mock-up was conducted using the proposed method (see Section 2). To maximize the delivered power with the available PAs (max 10 W each), staying within the safe operating areas of the electronic devices composing the chain, only the phase coefficients of the array ($\varphi_{n}$) were taken into account during the optimization process. A feedback control system was used to lock the optimized phases and the constant amplitudes provided to the antennas to the desired values for the entire heating session (130 min), with a maximum error lower than 0.3° for the phase and lower than 0.04 dBm for the amplitude. As a result, each antenna in the array delivered approximately 7.5 W for a total output power of 60 W for the entire array applicator.

The optimized phases along with the simulated and experimentally measured magnitude of the active reflection coefficients are reported in Table 4. A noteworthy level of agreement was observed between the simulated and experimental values, affirming the reliability of the model implemented in COMSOL in accurately reproducing the behavior of the realized prototype. Using the proposed approach, we achieved active reflection coefficients that were almost all below the −10 dB threshold, as shown in Table 4. The simulated SAR profile corresponding to the optimized antenna phases (Table 4) for a total input power $P_{0}=60$ W provided an average SAR of 33 W/kg in the tumor target region and 10 W/kg in the surrounding healthy tissues.

**Figure 10:**
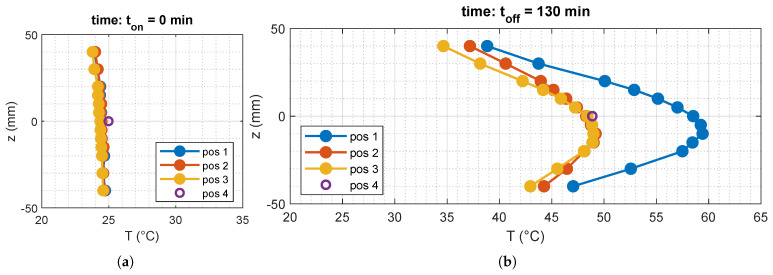
Temperatures recorded by the FOS arrays at (**a**) the beginning and at (**b**) the end of the heating session.

**Figure 11:**
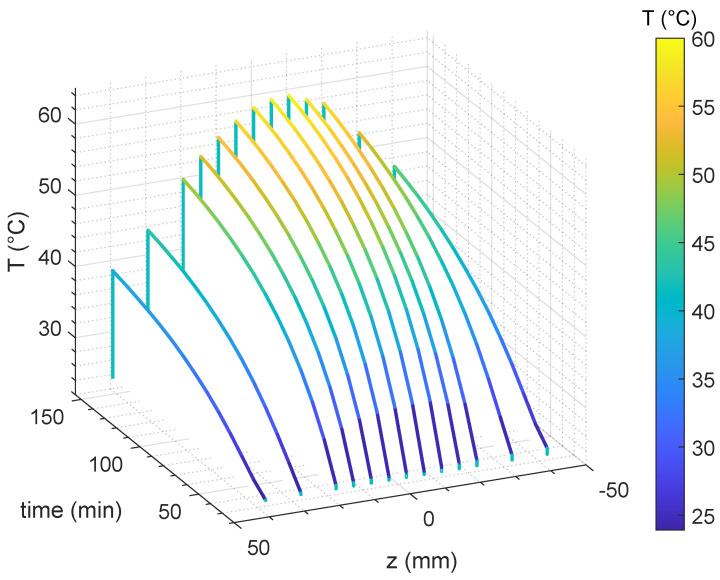
Temperature values read by the array of FBG sensors passing through the tumor target (array 1) as a function of time and of the *z*-coordinate.

The temperature measurements recorded at the start and at the end of the 130-minute heating session are illustrated in Figure 10a and Figure 10b, respectively. We observed that the duration of the heating session was longer than commonly encountered in the clinic as it was necessary to achieve meaningful temperature elevation within the tumor target region with the powers available in our laboratory setup. The maximum available power output was limited to 10 W per antenna, which is significantly lower than the power levels used in clinical hyperthermia setups, which typically operate at higher outputs and last 60–75 min [48]. Additionally, our experimental conditions required starting at room temperature, unlike clinical treatments, which begin at basal body temperature. It is important to emphasize that these experimental constraints did not compromise the validity of the proposed method or its ability to achieve precise energy deposition. This is demonstrated by the measurements depicted in Figure 10b, which confirm accurate radiation focusing on the tumor target region, as indicated by the observed temperature changes of array 1 (array passing tumor, see Figure 9a). A detailed overview of the temperature increases in array 1 is provided in Figure 11, where the measured temperature profiles are reported over time and along the *z*-coordinate.

To simulate the heating session, a heat transfer study was introduced into the in silico counterpart of the realized mock-up implemented in COMSOL Multiphysics and is described in Section 3.1. The used heat transfer model follows Pennes’ Bioheat Equation [49] and thermal boundary conditions were applied to account for the interactions of the neck phantom with the surrounding environment [50]. The convective heat flux boundary condition was used at the following interfaces: phantom upper boundary–air, phantom lower boundary–air, phantom lateral walls–waterbolus. Initial temperatures and external reference temperatures were set according to the measurements performed during the heating session.

**Figure 12:**
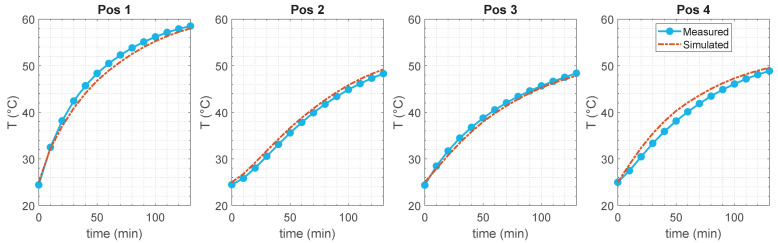
Measured temperatures and simulated values as a function of time at the *z*-coordinate corresponding to the tumor center ($z=z_{t}=0$ mm).

As evidence of the agreement between the mock-up and its in silico counterpart, the measured and simulated temperatures are shown as a function of time at the tumor center ($z=z_{t}=0$ mm) in Figure 12. This figure also effectively demonstrates the success of our method in achieving a favorable temperature increment in the tumor region during the heating session.

## 4. Conclusions

In this study, we introduced and demonstrated a novel approach to optimization for patient-specific hyperthermia planning in which array impedance matching is guaranteed. Our proposed optimization approach demonstrated improvements in antenna performance during HT treatments, without altering the required spatial power deposition recommended performance. The method ensures that almost all active reflection coefficients remain below the −10 dB threshold, which is important for optimizing power coupling and maintaining electronic stability. By incorporating active reflection coefficient constraints, we achieved satisfactory SAR focusing on the tumor target while ensuring that the electronic system remained fully operational thanks to controlled mismatching. The results show that our method significantly improves antenna matching without compromising the HTQ, achieving values within the recommended limits. Furthermore, the proposed approach is not confined to SAR-based optimizations; it can also be adapted for temperature-based methods.

## Figures and Tables

**Table 1:** Dielectric and thermal properties of the materials used in the mock-up at f=434 MHz.

Material	*ρ* (kg/m^3^)	$\varepsilon_{r}$ (-)	σ (S/m)	*k* (W/(m °C))	$C_{p}$ (kJ/(kg °C))
PMMA	1410	2.33 *	$10^{-4}$ *	0.39	1.4
Water	997	79.53 *	0.047 *	0.6	4.18
Muscle phantom	1138.27±3.56 *	58.09±0.98 *	0.91±0.04 *	0.50±0.01 *	3.20±0.07 *

* Experimentally measured.

**Table 2:** Antenna feeding coefficients ($\xi_{n},\varphi_{n}$) and corresponding magnitude of the simulated active reflection coefficients $\left(\left|\Gamma_{n,sim}^{a}\right|\right)$.

	Standard Optimization Approach			Proposed Optimization Approach		
n	$\xi_{n}$ (-)	$\varphi_{n}$ (°)	$\left|\Gamma_{n,\mathrm{sim}}^{a}\right|$ (dB)	$\xi_{n}$ (-)	$\varphi_{n}$ (°)	$\left|\Gamma_{n,\mathrm{sim}}^{a}\right|$ (dB)
1	0.69	0	−9.85	0.73	0	−12.76
2	0.50	73.41	−5.13	0.59	87.46	−13.38
3	0.50	76.24	−4.69	0.64	147.32	−10.00
4	0.50	0.00	−7.62	0.66	151.63	−10.00
5	0.50	47.04	−4.47	0.50	111.66	−10.00
6	0.79	1.89	−12.57	0.72	11.86	−15.73
7	1.00	−44.49	−9.69	0.99	−24.50	−12.32
8	1.00	−50.22	−9.53	1.00	−27.97	−12.00

**Table 3:** Comparison of the considered optimization approaches and their properties.

	Standard (HTQ)	Proposed $\left(F\right)$
Population size	100	100
No. of iterations	214	368
Total time (s)	139	203
HTQ (final)	0.906	0.959

**Table 4:** Antenna phases optimized using the proposed cost function $\left(F\right)$ and comparison of the simulated and measured magnitude of the active reflection coefficients.

*n*	1	2	3	4	5	6	7	8
$\varphi_{n}$ (°)	0	79.83	156.72	−156.06	−142.04	0	−43.86	−43.11
$\left|\Gamma_{n,sim}^{a}\right|$ (dB)	−18.42	−10.60	−11.90	−12.44	−10.81	−16.53	−16.23	−20.02
$\left|\Gamma_{n,exp}^{a}\right|$ (dB)	−15.99	−7.58	−10.11	−15.59	−18.24	−11.34	−23.89	−25.57

## Data Availability

No new data were created or analyzed in this study. Data sharing is not applicable to this article.
